# Use of trastuzumab in treating breast cancer during pregnancy: a systematic review and meta-analysis

**DOI:** 10.1186/s12905-021-01301-9

**Published:** 2021-04-21

**Authors:** Lin-Yu Xia, Qing-Lin Hu, Qing Zhou

**Affiliations:** Department of Thyroid and Breast Surgery, The First Affiliated Hospital of Chengdu Medical College, 278 Baoguang Avenue Middle Section, Xindu District, Chengdu City, 610500 Sichuan Province China

**Keywords:** Breast cancer, Pregnancy, Trastuzumab, Complication, Safety

## Abstract

**Background:**

Trastuzumab is currently the standard treatment for human epidermal growth factor receptor 2 (HER2)-positive breast cancer. However, it is not recommended for HER2-positive breast cancer patients during pregnancy as it may jeopardize safety of the fetus. Nevertheless, there is evidence that fetuses exposed to trastuzumab in early stages of pregnancy remain healthy

**Methods:**

To evaluate the possible effects of trastuzumab on fetus and provide evidence on the safety of trastuzumab in early pregnancy in HER2-positive breast cancer patients, we analyzed 22 studies involving 22 pregnant women and 23 fetuses.

**Results:**

Based on the meta-analysis, the gestational week of exposure to trastuzumab is 0–34 weeks, the average duration of use is 17 weeks, and the average gestational week of delivery is 34.3 weeks. Complications occurred in 77.27% of patients during pregnancy and 56.52% of newborns。The main complication during pregnancy was anhydramnios (68.18%), while the main complications at birth were Respiratory distress or tachypnea (30%). After an average of 25.28 months of follow-up, 17.39% (4/23) of the children died. There was no complication during pregnancy or at birth in patients treated with trastuzumab during early pregnancy (*P* = 0.043). Patients older than 30 who received trastuzumab during pregnancy were more likely to have neonatal complications (OR = 7.778, 95%CI = 1.2–50.424, *P* = 0.04).

**Conclusion:**

These results suggest that trastuzumab use during pregnancy can cause pregnancy,fetal and newborn complications. However, exposed to trastuzumab only in the first trimester are less likely to have pregnancy and fetal complications. Patients with gestational age below 30 years are less likely to have neonatal complications after trastuzumab during pregnancy. Terminating pregnancy should not be the only option for such patients. But more evidence is needed to verify this conclusion.

## Background

Breast cancer in pregnancy is a unique type of breast malignancy accounting for 2.8% of all breast cancer [1]. It is among the most common cancers in pregnant women, second only to melanoma, lymphoma and leukemia [2]. Globally, its occurrence is approximately 1 in 3000–10,000 pregnancies [3]. Treatment of breast cancer in pregnancy is challenging owing to the need for prompt intervention while safeguarding fetal safety. HER2 has been shown to be overexpressed in breast cancer patients during pregnancy compared to their nonpregnant counterparts [4]. Moreover, approximately one third of breast cancer in pregnancy cases are HER2-positive [5]. Trastuzumab is the first-line treatment for HER2-positive breast cancer, with good results in reducing the risk of relapse and improving overall survival. However, due to its association with embryo-fetal toxicity, trastuzumab is currently not recommended for breast cancer in pregnancy therapy in the National Comprehensive Cancer Network (NCCN) and the European Society for Medical Oncology (ESMO) clinical practice guidelines. However, the use of trastuzumab does not cause amenorrhea in women [6]. Even if the patients who use with trastuzumab therapy are advised to pay attention to contraception, unexpected pregnancy also exists. Under this circumstance, even if trastuzumab is stopped immediately, it is not clear what kind of pregnancy outcomes may be caused by exposure to trastuzumab in early pregnancy.

Currently, there are many safety reports on the use of chemotherapy drugs during pregnancy, but data on the safety of trastuzumab use during pregnancy are limited. A growing body of evidence suggests that fetuses exposed to trastuzumab in the early stages of pregnancy are born healthy, without any congenital malformations [1, 7, 8]. Evidence of cause and effect is paramount when deciding either to terminate or retain the pregnancy. The patient, the oncologist and health care workers need a common understanding on the effects of trastuzumab on the fetus and the pregnancy. Therefore, there is an urgent need to provide clinical guidance on how to manage these patients, especially those willing to retain pregnancy.

This work reviewed previous literature and collected a total of 22 articles, involving 22 pregnant women and 23 fetuses. By evaluating fetal safety in pregnant, breast cancer patients on trastuzumab therapy, we hope to shed light on safety of using trastuzumab in treating breast cancer during pregnancy.

## Methods

### Search strategy

This systematic review was carried out in accordance with the Preferred Reporting Items for Systematic reviews and Meta-Analyses (PRISMA) statement. A comprehensive search was performed in MEDLINE/PubMed and Embase databases for the period up to February 18, 2020. Articles were obtained using the keywords; breast neoplasms OR (breast and neoplasms), or breast cancer OR (breast and cancer), OR breast carcinoma, OR (breast and carcinoma) AND pregnancy AND (trastuzumab OR herceptin). A total of 56 articles were retrieved across all geographical locations and languages. Currently, there is limited information on the safe use of trastuzumab to treat breast cancer during pregnancy. Most data on trastuzumab use during pregnancy were obtained from case reports or small sample reviews. However, no prospective studies have been conducted. This is partly due to low incidence of breast cancer during pregnancy, and because trastuzumab is not recommended for use in the NCCN and ESMO clinical practice guidelines.

### Inclusion and exclusion criteria

Retrieved literature was screened using a predetermined inclusion and exclusion criteria. The inclusion criteria contained; (1) breast cancer patients were exposed to trastuzumab during pregnancy, (2) studies including at least one of the main outcomes of our study. The exclusion criteria contained; (1) spontaneous abortion, (2) voluntary termination of pregnancy.

### Data abstraction and outcome measures

The following data were collected from the eligible studies using an abstraction form: name of first author, stage of the tumor and receptor, treatment during pregnancy, age at conception, gestational age for diagnosis of pregnancy, drug use frequency, dose, gestational age of exposure to trastuzumab, exposed to other chemotherapy drugs and exposed gestational weeks, pregnancy and fetal complications, gestational weeks and mode of delivery, neonatal weight, apgar score, complications and follow-up results. The main outcomes of our study were pregnancy, fetal and newborn complications, and follow-up results of neonatal health.

### Statistical analysis

We use Fisher's exact test to analyze the possible influencing factors of pregnancy and fetal complications, new born complications and child death. The OR value was calculated to analyze the correlation between the influencing factors and complications. Statistical analysis was performed with SPSS version 20.0 statistical software. All *P* values were two-sided with a *P* < 0.05 considered statistically significant.

## Results

**Fig. 1:**
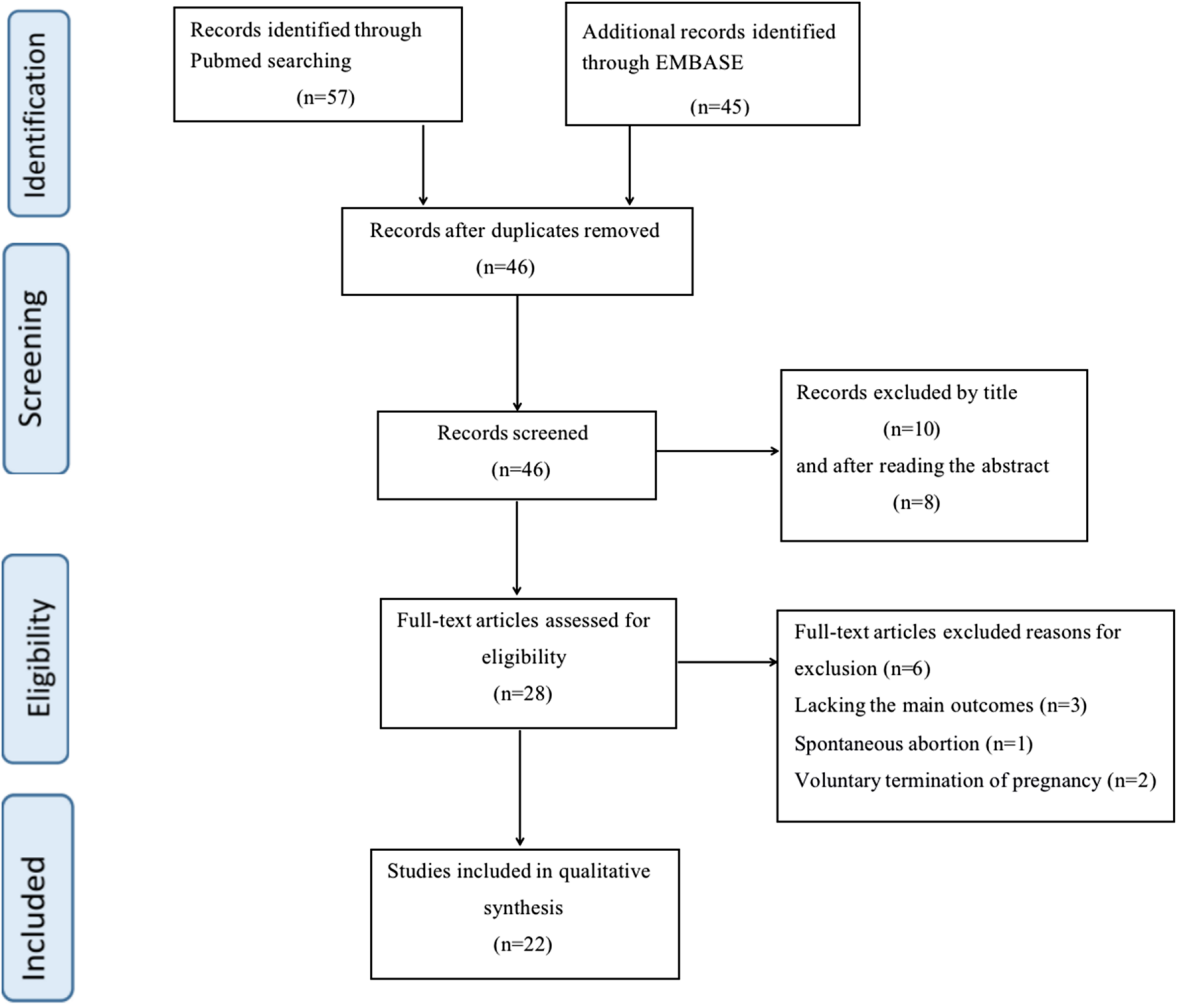
Flowchart explaining the study selection

After screening, 22 articles that satisfied the inclusion criteria were selected for meta-analysis [1, 7–27]. Figure 1 shows the process of selecting the included studies. In the 22 cases, 2 cases did not report the gestational week at the time of confirmation of pregnancy [12, 23]. Among the remaining 20 patients who reported the gestational week at the time of pregnancy confirmation, 14 (70%) patients chose to continue using trastuzumab after pregnancy confirmation [7, 10, 13–17, 19, 21, 22, 24–27]. Among the 22 patients, 20 patients reported the the status of the receptor,15 of 20 (75%) were hormone receptor negative [1, 7–12, 14, 15, 17, 20–22, 25, 27] while 5 (25%) were hormone receptor positive [13, 14, 18, 19, 24]. Age of the sampled pregnant patients ranged from 27 to 38 years, with an average age of 31.82 years. Gestational weeks of delivery ranged from 27 to 39 weeks, with an average of 34.3 weeks. In 13 (61.9%) patients, cesarean section was used in delivery while 8 (38.1%) patients delivered through spontaneous labor. Delivery method was not reported in one of the patients [19]. Table 1 illustrates the Patients and tumours’ characteristics, treatments and outcomes.

**Table 1:** Patients and tumours’ characteristics, treatments and outcomes

Characteristic	Goodyer et al. [7]	Waterston et al. [1]	Azim Jr et al. [8]	Min et al. [9]	Pant et al. [10]
Stage	IV	NR	IIB	NR	IV
Treatment	Trastuzumab	Trastuzumab	Trastuzumab	Trastuzumab	Trastuzumab
Receptor (ER\PR\HER-2)	(−)(−)(+)	(−)(−)(+)	(−)(−)(+)	(−)(−)(+)	(−)(−)(+)
Age at pregnancy (y)	33	30	29	32	32
GA (week)	14	3	3	17	14
Trastuzumab					
Schedule (weeks)	1	3	3	3	3
Total dose	NR	1259 mg	NR	600 mg	4200 mg
GA (week)	14–29	0–3	0–1	0–17	0–32
Chemotherapy	No	No	No	No	No
Pregnancy/fetal complication	No	No	No	No	Anhydramnios
Delivery					
Weeks	29	NR	39	38 + 5	32 + 1
Mode	cs	Vaginal	cs	Vaginal	Vaginal
New born					
Weight (g)	1220	NR	3550	3570	1810
Apgar scores	NR	NR	NR	9, 10	normal
Complications	Respiratory distress syndrome	No	No	No	No
Follow-up (months)	Minimal tightness of the left Achilles tendon at 36 months	NR	Healthy at 14 months	NR	healthy at 60 months

Characteristic	Pianca et al. [11]	Rasenack et al. [12]	Bader et al. [13]	Mandrawa et al. [14]	Roberts et al. [15]
Stage	IIA	IV	IV	IV	IIB
Treatment	Trastuzumab	Trastuzumab	46 Gy (cervical vertebra) with shielding + trastuzumab + paclitaxel	Trastuzumab	Trastuzumab
Receptor (ER\PR\HER-2)	(−)(−)(+)	(−)(−)(+)	(−)(+)(+)	(−)(−)(+)	(−)(−)(+)
Age at pregnancy (y)	30	29	38	28	36
GA (week)	28	NR	17	12	17
Trastuzumab					
Schedule (weeks)	3	3	3	3	3
Total dose	NR	NR	NR	3510 mg	NR
GA (week)	0–28	0–24	25–28	0–27	4–21
Chemotherapy	No	No	Paclitaxel (25 week)	No	No
Pregnancy/fetal complication	Anhydramnios	Anhydramnios	Anhydramnios, fetal renal failure	Anhydramnios	Decline of cardiac ejection fraction
Delivery					
Weeks	37	36	32	37	37
Mode	Vaginal	CS	CS	Vaginal	Vaginal
New born					
Weight (g)	2535	NR	1460	3060	3200
Apgar scores	4, 8	NR	NR	NR	NR
Complications	No	No	Signs of bacterial sepsis (hypotension, transient renal failure, respiratory failure, positive laboratory findings	Transient tachypnoea at birth	Mild transient tachypnoea
Follow-up (months)	Healthy at 84 months	Healthy at 60 months	Healthy at 3 months	Healthy at 28 months	NR

Characteristic	Witzel et al. [16]	Shrim et al. [17]	Beale et al. [18]	Warraich et al. [19]	
Stage	IV	IV	NR	NR	
Treatment	Trastuzumab	Trastuzumab	Trastuzumab + tamoxifen	Trastuzumab + tamoxifen + goserelin	
Receptor (ER\PR\HER-2)	(+)(+)(+)	(−)(−)(+)	(+)(+)(+)	(+)(+)(+)	
Age at pregnancy (y)	34	32	29	35	
GA (week)	23	5	23	7	
Trastuzumab					
Schedule (weeks)	3	3	3	3	
Total dose	NR	3200 mg	NR	3675 mg	
GA (week)	0–26	0–24	0–22	7–31	
Chemotherapy	No	No	No	No	
Pregnancy/fetal complication	Oligohydramnios and vaginal bleeding	Asymptomatic low ejection fraction (weeks 18, 24)	Anhydramnios	Anhydramnios	
Delivery					
Weeks	27	37	31	34	
Mode	CS	CS	CS	NR	
New born					
Weight (g)	1015	2600	1590 and 1705	NR	
Apgar scores	8, 7	9, 10	5, 8 and 8, 10	NR	
Complications	Respiratory failure, strong capillary link syndrome, persisting infections, necrotizing enterocolitis	Transient tachypnea at birth	Twin A: chronic lung disease and renal failure, Twin B: creatinine elevation and respiratory distress syndrome at birth	Severe pulmonary hypoplasia and atelectasis	
Follow-up (months)	Dead at 5.25 months	Healthy at 2 months	Twin A:Dead from respiratory arrest at 0.25 months, TwinB:Healthy at 0.25 months	Dead within 40 min at birth	

Characteristic	Watson et al. [20]	Fanale et al. [21]	Sekar et al. [22]	Weber et al. [23]	
Stage	IIIB	IV	IV	IV	
Treatment	Trastuzumab	Vinorelbine + trastuzumab	Docetaxel + trastuzumab	Trastuzumab	
Receptor (ER\PR\HER-2)	(−)(−)(+)	(−)(−)(+)	(−)(−)(+)	NR	
Age at pregnancy (y)	28	27	28	32	
GA (week)	23	27	20	NR	
Trastuzumab					
Schedule (weeks)	3	1	3	3	
Total dose	3480 mg	2700 mg	1385 mg	NR	
GA (week)	0–20	27–34	23–27	0–23	
Chemotherapy	No	Vinorelbine (at 27 week)	Docetaxel (at 23 week)	No	
Pregnancy/fetal complication	Anhydramnios and small fetal bladder (23 weeks)	Anhydramnios	Anhydramnios	Anhydramnios	
Delivery					
Weeks	37	34	36	27	
Mode	Vaginal	Vaginal	CS	CS	
New born					
Weight (g)	2960	2580	2230	NR	
Apgar scores	8, 9	9, 9	7, 9	NR	
Complications	No	No	No	Multiple prematurity related problems at birth	
Follow-up (months)	Healthy at 6 months	Healthy at 6 months	NR	Dead at 4 months	

Characteristic	Gottschalk et al. [24]	El-Safadi et al. [25]	Andrade et al. [26]	Aktoz et al. [27]	
Stage	NR	IV	III	IV	
Treatment	Docetaxel + carboplatin + trastuzumab	Vinorelbine + trastuzumab + ibandronate	Trastuzumab	Docetaxel + trastuzumab	
Receptor (ER\PR\HER-2)	(+)(+)(+)	(−)(−)(+)	NR	(−)(−)(+)	
Age at pregnancy (y)	38	32	31	37	
GA (week)	11	29	9	22	
Trastuzumab					
Schedule (weeks)	1	3	3	3	
Total dose	NR	NR	4400 mg	2293 mg	
GA (week)	14–17	30–33	0–32	22–34	
Chemotherapy	Docetaxel + carboplatin (at 17 week)	Vinorelbine + ibandronate (at 30 week)	No	Docetaxel (at 22 week)	
Pregnancy/fetal complication	Anhydramnios, intrauterine growth restriction, fetal renal insufficiency	Anhydramnios	Anhydramnios	No	
Delivery					
Weeks	33	33	32^+2^	35^+3^	
Mode	CS	CS	CS	CS	
New born					
Weight (g)	NR	1990	1655	2850	
Apgar scores	NR	8, 9	4, 10	8, 8	
Complications	Dystrophic premature neonate at birth	No	Respiratory distress syndrome and a pulmonary infection	Transient renal insufficiency	
Follow-up (months)	NR	Healthy at 12 months	Healthy at 84 months	Healthy at 5 days	

*GA* gestational age, *NR* not reported, *CS* cesarean section

**Fig. 2:**
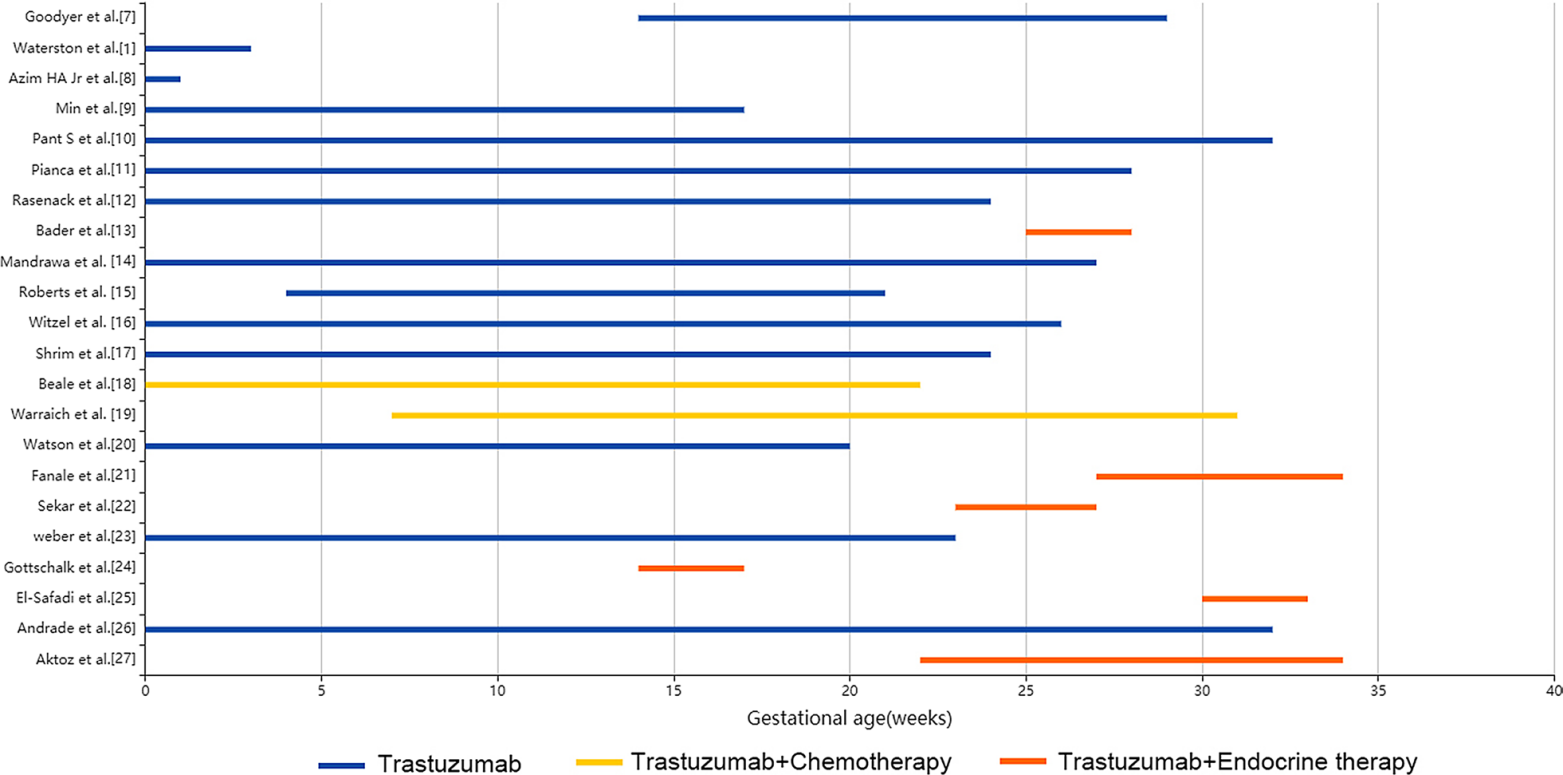
Gestational age of patients receiving different treatments

The gestational age of exposure to trastuzumab was 0–34 weeks. The average duration of use was 17 weeks, and the average total dose of trastuzumab was 2791 mg. It is worth noting that trastuzumab is not completely used alone. 2 pregnant women and 3 fetuses used a combination of trastuzumab and endocrine therapy. One of the pregnant women was treated with tamoxifen endocrine therapy [18], and the other was treated with tamoxifen + goserelin [19]. 6 pregnant women and 6 fetuses used a combination of trastuzumab and chemotherapy drugs [13, 21, 22, 24, 25, 42] (Fig. 2).

**Fig. 3:**
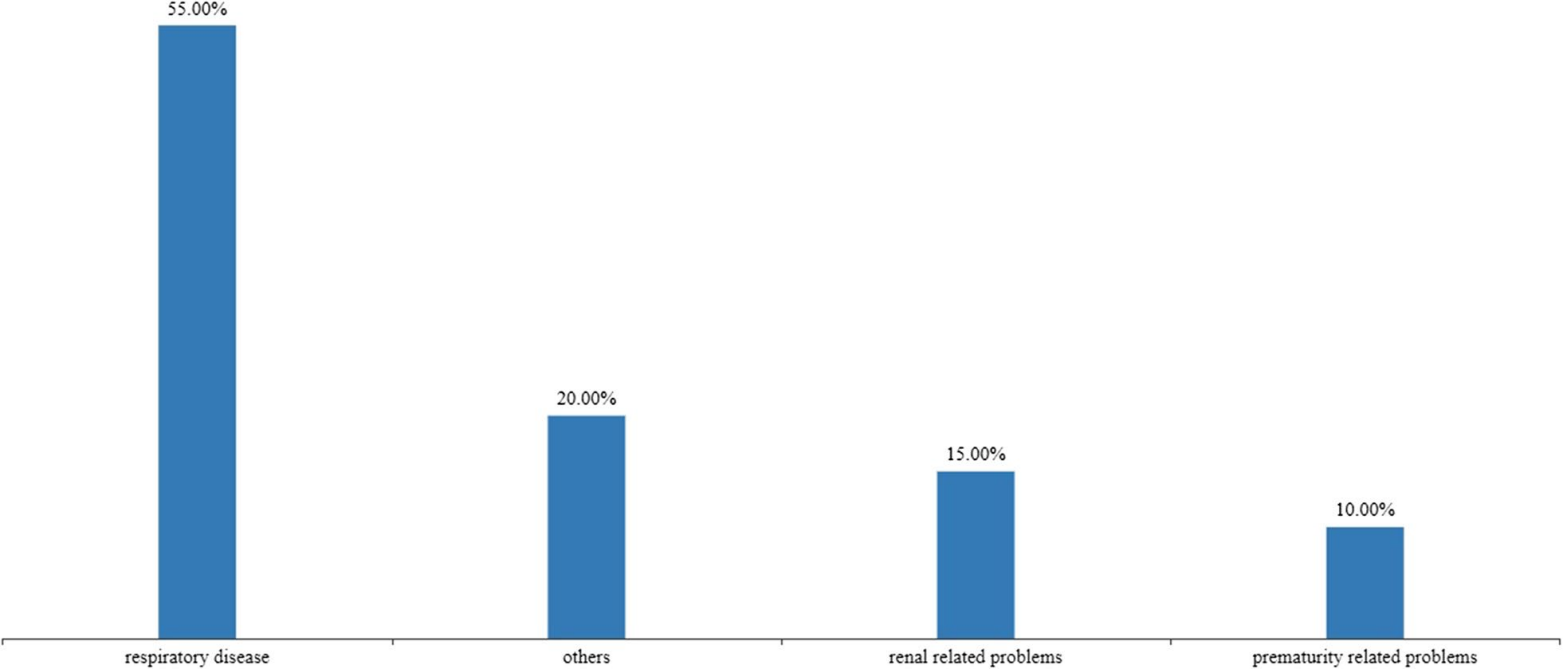
Incidence of various complications in neonates

Complications occurred in 77.27% of patients during pregnancy. Amniotic fluid reduction is the most common, with an incidence rate of 68.18% (15/22) [10–14, 16, 18–26], and the remaining 9.09% (2/22) patients had a decreased cardiac ejection fraction [15, 17]. After birth, the average apgar score of the newborn were 7,9 and the average birth weight was 2310 g. 39.13% (9/23) of the newborns were healthy, while 60.87% (14/23) of the newborns had complications [1, 8–12, 20–22, 25–27]. The most common neonatal complications were respiratory related problems, including transient tachypnoea and respiratory distress syndrome [7, 14, 15, 17, 18, 26] (Fig. 3). After an average follow-up of 25.28 months, 17.39% (4 / 23) of the children died [16, 18, 19, 23]. All children who died suffer from serious complications after birth.

Trastuzumab only exposed in early pregnancy had fewer pregnancy and fetus related complications than other periods (*P* = 0.043), but there was no significant difference in the incidence of neonatal complication (*P* = 0.178). Patients with gestational age greater than 30 years were more likely to have neonatal complications after trastuzumab during pregnancy (OR = 7.778, 95%CI = 1.2–50.424, *P* = 0.04). Pregnancy and fetal complications,neonatal complications and child deaths were not statistically different in terms of tumor stage,trastuzumab schedule, exposed time, total dose of trastuzumab, combined endocrine therapy and combined chemotherapy (Table 2).

**Table 2:** Analysis of the influencing factors of pregnancy, neonatal complications and child death

Characteristic		Pregnancy/fetal			95%CI	*P*	New born complication			95%CI	*P*	Child deaths			95%CI	*P*
		Yes	No	OR			Yes	No	OR			Yes	No	OR		
Age (years)	> 30	10	3	0.95	0.125–7.275	1	10	3	7.78	1.2–50.424	0.04	3	7	3	0.248–36.325	0.588
	≤ 30	7	2				3	7				1	7			
Tumor stage	IV	10	2	3.33	0.319–34.830	0.538	7	5	2.1	0.251–17.594	0.62	2	9	NA	NA	1
	I–III	3	2				2	3				0	4			
Exposed gestational stage	1st trim	0	2	NA	NA	0.043	0	2	NA	NA	0.178	0	2	NA	NA	1
	others	17	3				13	8				2	14			
Trastuzumab schedule	3 weeks	15	4	1.88	0.134–26.320	1	11	9	0.61	0.047–7.882	1	4	12	NA	NA	1
	1 week	2	1				2	1				0	2			
Exposed time (weeks)	≤ 12	5	2	0.63	0.079–4.959	1	2	5	0.18	0.026–1.279	0.169	0	4	NA	NA	0.524
	> 12	12	3				11	5				4	10			
Total dose (mg)	≤ 3000	2	3	NA	NA	0.061	1	4	0.13	0.008–1.998	0.242	0	2	NA	NA	1
	> 3000	6	0				4	2				1	5			
Chemotherapy	Yes	5	1	1.67	0.147–18.874	1	3	3	0.7	0.108–4.538	1	0	4	NA	NA	0.524
	No	12	4				10	7				4	10			
Endocrine therapy	Yes	2	0	NA	NA	1	3	0	NA	NA	0.526	2	1	13	0.771–219.107	0.108
	No	15	5				12	8				2	13			

*1st trim.* the first trimester, *NA* not applicable

## Discussion

Breast cancer in pregnancy is characterized by a low hormone receptor-positive, a higher Ki-67 nuclear antigen index and overexpression of HER2 [28–30]. Moreover, it is more invasive than breast cancer in patients who are not pregnant. From this meta-analysis, 73.7% of patients were hormone receptor-negative, further supporting current evidence on hormone receptor status of breast cancer in pregnancy. The average gestational age at delivery was 34.3 weeks, suggesting that pregnant women exposed to trastuzumab are likely to give birth prematurely, possibly due to decreased amniotic fluid. Furthermore, hypohydramnios may be associated with potential placental dysfunction and decreased feto-placental circulation [31]. This may lead to placental ischemia, and Placental ischemic disease may be an important cause of premature delivery [32].

Trastuzumab is an Immunoglobulin G (IgG) humanized monoclonal antibody (McAb) used as the first line of treatment for HER2-positive breast cancer. IgG is the only monoclonal antibody that can cross the placental barrier to block HER2 protein. HER2 is known to play an important role in embryonic development. Current evidence indicates that trastuzumab does not have a direct adverse effect on the fetus, but may increase the risk of fetal morbidity and mortality by reducing amniotic fluid. Trastuzumab is associated with the production of vascular endothelial growth factor which regulates production and reabsorption of amniotic fluid by changing the permeability of fetal membranes. This may cause oligohydramnios. Another possible mechanism is that trastuzumab may affect aquaporin channels in the renal tubular basement membrane, resulting in fetal renal insufficiency and oligohydramnios [22]. Amniotic fluid loss was significantly correlated with trastuzumab exposure during pregnancy trimesters. In this study, amniotic fluid loss rate was 0% in early pregnancy and 85% in the middle and late pregnancy, indicating that the risk of amniotic fluid loss in early pregnancy is very low. Studies have shown that the degree of oligohydramnios is related to the length of exposure time of trastuzumab [8], which has been demonstrated by reversal of oligohydramnios through discontinuation of trastuzumab therapy. For instance, Watson observed that the amniotic fluid index increased slowly after discontinuing use of trastuzumab [20]. Through close patients follow ups, Sekar reported a slow recovery in amniotic fluid volume after discontinuation of trastuzumab therapy [22]. The shorter the exposure time, the faster the amniotic fluid returns to its normal volume and the lesser the effect on fetal growth. This suggests that long-term intrauterine fetal exposure to trastuzumab treatment could significantly increase occurrence of serious adverse effects on the fetus. Therefore, if trastuzumab must be used during pregnancy in consideration of the disease of the mother, short-term use should be chosen as far as possible and long-term exposure to trastuzumab should be avoided.

During pregnancy, 9.09% of the sampled patients had reduced ejection fraction [15, 17]. The exact mechanism of cardiotoxicity induced by trastuzumab is still unclear [33]. Preliminary studies have shown that HER2 receptor can inhibit cardiomyocyte apoptosis, reduce Reactive Oxygen Species release and enhance endothelial Nitric Oxide Synthase expression through HER4 receptor, which plays a crucial role in maintaining the myocardium. Inhibition of HER2 receptor may cause myocardial damage [34]. However, trastuzumab-mediated myocardial damage is reversible upon discontinuation of treatment. Human epidermal growth factor is essential for embryonic heart development. According to Lee [35], mutant mice lacking human epidermal growth factor had a high embryo mortality rate, presumably due to the absence of cardiac trabeculae. But no studies have reported trastuzumab to have a damaging effect on fetal heart. This may be due to the fact that trastuzumab is rarely used during pregnancy. From our meta-analysis, there were no records of fetal or neonatal heart dysplasia during pregnancy.

HER2 is strongly expressed in fetal renal epithelium, inducing DNA synthesis and mitotic activity in renal cells. Studies have shown that expression of epidermal growth factor receptor on offspring kidneys is significantly higher than in adult kidneys [14]. Therefore, blocking of epidermal growth factor receptor in fetal kidneys by trastuzumab may impair renal function. Some scholars studied the use of monoclonal antibodies to block epidermal growth factor receptor in fetal kidneys during the second trimester of pregnancy, reporting a decrease in renal cell proliferation [36, 37]. We report here three cases of neonatal renal impairment, with an incidence rate of 13.04%(3/23) [13, 18, 27]. Trastuzumab exposure time extended from early to mid-pregnancy. Two newborns died after birth while one survived after transient renal failure. These results suggest that prenatal exposure to trastuzumab may result in fetal renal impairment.

After delivery, 47.83% (11/23) of the newborns experienced respiratory disease. Fetal lung development is strongly linked to fetal thoracic volume and fetal respiratory movement, while pulmonary dysfunction is most often associated with amniotic fluid loss [38]. The fetus inhales amniotic fluid into the lungs through respiratory movement, causing pulmonary dilatation, the main driver of lung development [39]. Too little amniotic fluid results in reduced chest cavity, inhibits growth of the fetal lung, and leads to lung dysplasia [40, 41]. Amniotic fluid loss is the most common complication of trastuzumab use during pregnancy, so the incidence of respiratory diseases is not low. It is therefore important to monitor the amniotic fluid regularly.

Unlike other chemotherapy drugs, the use of trastuzumab as treatment for breast cancer may be safe in early pregnancy stages. This review and meta-analysis concludes that use of trastuzumab in early pregnancy has no adverse effects, with a zero chance of developing complications during pregnancy and birth. This might be as a result of low metastasis of McAb in early pregnancy [12]. Monoclonal antibodies have a high molecular weight and can only be transported through the placenta by binding to a specific neonatal Fc receptor for IgG (FcRn). FcRn receptor is undetachable in the early pregnant mothers, therefore, early pregnancy monoclonal antibodies are rarely transported to the embryo via the placenta. The concentration of IgG in the fetus begin to increase from 18 weeks of gestation, and increase rapidly at 24–26 weeks of gestation [42]. Consequently, from the second trimester of pregnancy, the risk of fetal birth complications caused by trastuzumab treatment is greatly increased. According to our analysis, fetuses and pregnant women in patients who received trastuzumab only in the first trimester were relatively safe, and termination of pregnancy is the safest choice for patients receiving trastuzumab in the second trimester of pregnancy, but it could also be considered in combination with the patient's age, exposure time, amniotic fluid volume, and the patient's willingness to continue the pregnancy. In late pregnancy, one option of avoiding the safety concerns associated with trastuzumab is through inducing premature delivery before embarking on a trastuzumab therapy for breast cancer.

Our research has some limitations. Considering that intrauterine exposure to trastuzumab can lead to fetal and neonatal complications, there are few reports on the use of trastuzumab during pregnancy. The evidence we can gather at present are all case reports,and only 22 cases, so there are few available data. It should be emphasized that only two patients used trastuzumab in early pregnancy, so we need to be cautious about our results. Secondly, not all the data can be obtained from the collected case reports, so we can not make further logistic regression for the data. Finally, there may be publication bias due to our analysis of only published research. Nevertheless, our research may be useful for patient's conceives while already undergoing trastuzumab therapy, and can provide some reference for them to decide whether or not to continue their pregnancy.

## Conclusion

Maternal and fetal health should be considered during diagnosis and treatment of breast cancer in pregnancy. In our study, the risk of severe complications as a result of trastuzumab use in early pregnancy is relatively low for patients no more than 30 years old. Routine use of trastuzumab is not recommended for HER2-positive breast cancer in pregnancy. However, for young patients with unexpected pregnancy who used trastuzumab only in the first trimester, they can choose to continue pregnancy,but should stop using trastuzumab in time and close monitoring of fetal health and regular monitoring of the amniotic fluid volume during pregnancy.

## Data Availability

All the data are available without restriction. Researchers can obtain data from the corresponding author.

## Abbreviations

HER2: Human epidermal growth factor receptor 2
IgG: Immunoglobulin G
McAb: Monoclonal antibody
FcRn: Neonatal Fc receptor
